# CSF-1 Overexpression Predicts Poor Prognosis in Upper Tract Urothelial Carcinomas

**DOI:** 10.1155/2019/2724948

**Published:** 2019-08-21

**Authors:** Wei-Chi Hsu, Yi-Chen Lee, Peir-In Liang, Lin-Li Chang, A-Mei Huang, Hui-Hui Lin, Wen-Jeng Wu, Ching-Chia Li, Wei-Ming Li, Jhen-Hao Jhan, Hung-Lung Ke

**Affiliations:** ^1^Graduate Institute of Medicine, College of Medicine, Kaohsiung Medical University, Kaohsiung, Taiwan; ^2^Department of Urology, Kaohsiung Medical University Hospital, Kaohsiung Medical University, Kaohsiung, Taiwan; ^3^Department of Anatomy, School of Medicine, College of Medicine, Kaohsiung Medical University, Kaohsiung, Taiwan; ^4^Department of Pathology, Kaohsiung Medical University Hospital, Kaohsiung Medical University, Kaohsiung, Taiwan; ^5^Department of Microbiology and Immunology, School of Medicine, College of Medicine, Kaohsiung Medical University, Kaohsiung, Taiwan; ^6^Department of Biochemistry, School of Medicine, College of Medicine, Kaohsiung Medical University, Kaohsiung, Taiwan; ^7^Graduate Institute of Clinical Medicine, College of Medicine, Kaohsiung Medical University, Kaohsiung, Taiwan; ^8^Department of Urology, School of Medicine, College of Medicine, Kaohsiung Medical University, Kaohsiung, Taiwan; ^9^Department of Urology, Ministry of Health and Welfare Pingtung Hospital, Pingtung, Taiwan

## Abstract

**Background:**

Colony-stimulating factor-1 (CSF-1) is a homodimeric glycoprotein. The main role of CSF-1 is as a hematopoietic growth factor that modulates proliferation, differentiation, and survival of macrophages. Moreover, CSF-1 has also been reported to be aberrantly expressed in several human cancers. However, the precise role of CSF-1 in upper tract urothelial carcinomas (UTUC) has not been studied. In this research, we examined the clinical significance of CSF-1 expression in UTUC.

**Materials and Methods:**

One hundred twelve cancer tissue samples of UTUC from patients were included in this study, and the other cohort of 35 UTUC were paired cancer-adjacent normal samples. CSF-1 expression was evaluated by immunohistochemistry, and the association of CSF-1 expression with different clinicopathological variables was analyzed.

**Results:**

CSF-1 expression was higher in UTUC than in the normal urothelium (*P* = 0.005). The CSF-1 expression was primarily localized in the nucleus and was significantly correlated with tumor size (*P* = 0.04) and patients who had a high stage (*P* < 0.001), distant metastasis (*P* = 0.006), recurrence (*P* = 0.003), and cancer death (*P* = 0.005). High CSF-1 expression was correlated with poor disease-free survival (*P* = 0.008) and cancer-specific survival (*P* = 0.001). Our results also used univariate and multivariable analyses, which found that high CSF-1 expression was an independent predictor of poor disease-free survival (hazard ratio = 2.56; *P* = 0.007) and cancer-specific survival (hazard ratio = 5.14; *P* = 0.022).

**Conclusions:**

Our findings indicate that the expression of CSF-1 is a potential prognostic marker for predicting patient survival and recurrence in UTUC.

## 1. Introduction

Urothelial carcinomas (UC) can be categorized into three groups: bladder (UCB), renal pelvis, and ureter [1]. Upper tract urothelial carcinomas (UTUC) includes both ureteral and renal pelvic tumors [2]. UTUC is a rare cancer with vastly different characteristics between eastern and western countries; e.g., the male-to-female ratio is 1 : 1.2 in Taiwanese UTUC patients [3] but the ratio of patients in western countries is reversed [4]. In western countries, the incidence of urothelial carcinomas presenting as UCB is 90-95% [5], while UTUC is rare, accounting for only 5-10% of all urothelial carcinomas [6–8]. However, the incidence of UTUC in Taiwan is markedly higher at 30% of all urothelial carcinomas [9]. It is probable that various genetic, environmental, and other risk factors lead to a higher incidence of UTUC in Taiwan [10, 11]. The main predicting factor for prognosis is the cancer stage [12]. However, even in the same pathological stage and with standard treatment, patients still have divergent prognoses. Our previous studies have demonstrated some possible prognostic biomarkers such as hypoxia-induced factor 1*α* (HIF-1*α*) [13], leptin receptor [14], and signal transducer and activator of transcription 3 (STAT3) [15] associated with UTUC. However, the exact molecular mechanism of UTUC progression is not widely understood, and therefore, no probable prognostic markers have been proven.

Colony-stimulating factor-1 (CSF-1), also called “macrophage colony-stimulating factor” (M-CSF), is an important hematopoietic growth factor. CSF-1 binds to its receptor—the colony-stimulating factor-1 receptor (CSF-1R/c-fms)—and regulates the survival, differentiation, and proliferation of the monocyte-macrophage lineage [16, 17]. Additionally, several studies reveal that CSF-1 can promote tumor cell progression, migration, invasion, and metastasis [18–21]. CSF-1 is produced by macrophages, fibroblasts, and epithelial cells and is also secreted by tumor cells. Overexpression of CSF-1 has been associated with several human cancers, including breast cancers [22, 23], renal cell carcinomas [24], and ovarian cancers [25]. Moreover, clinical studies have shown that high CSF-1 levels have been linked to a poor prognosis in pancreatic cancer [26], prostate cancer [27], colorectal cancer [28], and clear-cell renal cell carcinoma [29].

Because there is no published research investigating the role of CSF-1 in UTUC, we aim to examine the association between the clinicopathological behavior of UTUC and CSF-1 expression in cancer tissues.

## 2. Materials and Methods

### 2.1. Surgical Specimens and Clinicopathological Data

One hundred twelve formalin-fixed UTUC tissues and thirty-five paired noncancerous urothelial samples were obtained from the Department of Urology, Kaohsiung Medical University Hospital, from 1997 to 2006 as previously described [14, 15]. All samples were histologically confirmed to be UC. All patients were treated with nephroureterectomy and excision of the bladder cuff. Medical records were reviewed retrospectively and clinicopathological data were retrieved. A follow-up protocol was created according to the National Comprehensive Cancer Network (NCCN) guidelines. The median follow-up time was 40.39 months, and the range was between 1 and 136 months. Disease-free survival was calculated from the date of surgery to the date of UTUC recurrence. Cancer-specific survival was defined as the time from the date of surgery to the date of cancer death. The pathologic grade was classified according to the World Health Organization (WHO) histologic criteria, and tumor staging was determined according to the Union for International Cancer Control tumor-node-metastasis classification. The clinicopathological parameters were obtained by retrospectively reviewing medical records. An informed consent was provided to the patient and signed before surgery. The study protocol was reviewed and approved by the Institutional Review Board of Kaohsiung Medical University Hospital (KMUH-IRB-E(II)-20170070).

### 2.2. Immunohistochemical Staining of CSF-1

Four-micrometer-thick sections from paraffin-embedded blocks were cut onto precoated slides, followed by deparaffinization, rehydration, and antigen retrieval as previously described [14, 15]. Endogenous peroxidase was blocked per the manufacturer's protocol (Dako, Carpinteria, CA). The slides were incubated with an anti-CSF-1 monoclonal antibody (MABF191, Merck Millipore) at a 1 : 200 dilution at 4°C for 1 h. Primary antibodies were detected using the Dako ChemMate EnVision Kit (K5001, Dako, Carpinteria, CA). Finally, the slides were counterstained with hematoxylin and investigated by light microscopy.

### 2.3. Evaluation of Immunohistochemical Staining

Scoring for CSF-1-positive staining was decided based on the percentage of positively stained cells in 4 quantitative categories as previously described [14, 15]: score 1, <25% positive cells; score 2, 26% to 50% positive cells; score 3, 51% to 75% positive cells; and score 4, >76% positive cells. The cancer immunostaining was inspected by 2 qualified pathologists who were blinded to the clinical status of the patients. Any discrepancies in scoring between pathologists were jointly reviewed, and a concordance was reached.

### 2.4. Statistical Analysis

All statistical analyses were executed using the SPSS statistical package for PC (version 14.0, IBM, Armonk, NY) as previously described [14, 15]. As a representation of indicative CSF-1 levels, tumors with scores of 1 or 2 were categorized as low expression (i.e., <50% positively stained cells), whereas tumors with scores of 3 or 4 were categorized as high expression (i.e., >50% positively stained cells). A Wilcoxon singed-rank test was used to test the difference of the CSF-1 expression between UTUC and the tumor-adjacent normal urothelium. Fisher's and chi-square tests were used to analyze for associations between the CSF-1 expression and tumor size, tumor stage, tumor grade, gender, age, tumor side, lymphovascular invasion, distant metastasis, recurrence, and serum creatinine level. Survival curves were created using Kaplan-Meier estimates, and the importance of differences between curves was estimated using the log-rank test. In addition, hazard ratios (HRs) and 95% confidence intervals (CIs) calculated from univariate and multivariate Cox regression models were used to investigate the connection between clinicopathologic parameters and survival as previously described [14, 15]. *P* values less than 0.05 were regarded as statistically significant.

### 2.5. Cell Lines and Cell Culture

BFTC909, a human renal pelvis transitional cell line [30], was purchased from the Bioresource Collection and Research Center (BCRC, #60069, Taiwan). This cell line was cultured in Dulbecco's Modified Eagle Medium (DMEM) supplemented with 10% fetal bovine serum (FBS) and antibiotic-antimycotic (Gibco™) and incubated at 37°C, 5% CO_2_. UM-UC-14, a human transitional cell carcinoma of the renal pelvis, was purchased from the European Collection of Authenticated Cell Cultures (ECACC). This cell line was cultured in Eagle's Minimum Essential Medium (EMEM) supplemented with 10% FBS, 2 mM glutamine, 1% nonessential amino acids (NEAA), and antibiotic-antimycotic and incubated at 37°C, 5% CO_2_.

### 2.6. Immunofluorescence

BFTC909 and UM-UC-14 cell lines were seeded in a 35 mm Glass Bottom Dish (ibidi) and incubated at 37°C, 5% CO_2_. Immunofluorescence was performed using the Image-iT™ Fixation/Permeabilization Kit (Invitrogen™). We removed the culture medium from the cells and then performed cell fixation, permeabilization, and a blocking procedure per the manufacturer's protocol. After blocking, we aspirated the blocking solution and incubated the cells with an anti-CSF-1 monoclonal antibody (M-CSF Antibody (D-4), sc-365779, Santa Cruz) at a 1 : 50 dilution in blocking solution at 4°C overnight. The cells were then incubated with fluorescein isothiocyanate- (FITC-) conjugated secondary antibody diluted in phosphate-buffered saline (PBS) for 1 h at room temperature (protected from light). Next, the cells were incubated with DAPI (Thermo Scientific™) diluted in PBS for 10 min at room temperature in the dark. Finally, cells were mounted by ProLong™ Gold Antifade Mountant (Thermo Scientific™) and observed using a fluorescence microscope.

## 3. Results

### 3.1. CSF-1 Expression in Human UTUC and Nontumor Urothelial Tissues

**Figure 1:**
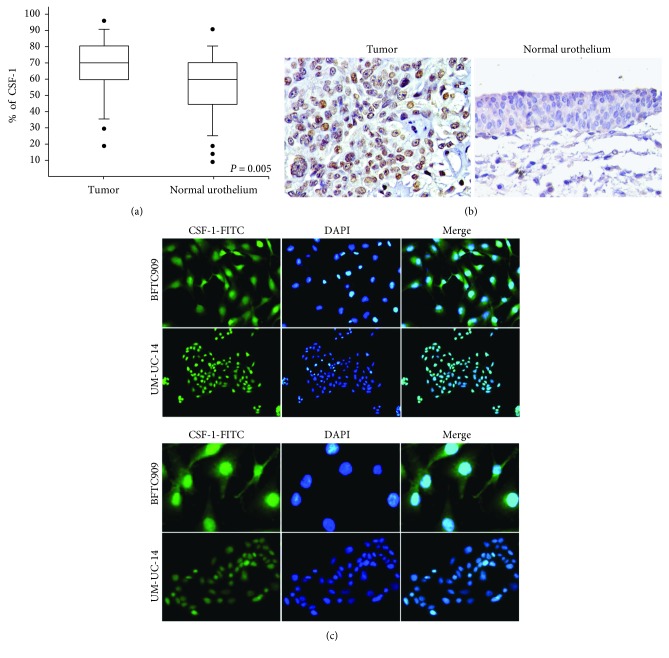
(a) Comparison with CSF-1 levels in 35 pairs of upper tract urothelial carcinomas (UTUC) and the corresponding cancer-adjacent normal tissues. The CSF-1 expression level was significantly higher in UTUC than in the normal urothelium (paired Wilcoxon signed-rank test, *P* = 0.005). (b) Immunohistochemistry staining for CSF-1 in UTUC and normal urothelium. (×200). (c) CSF-1 mainly localized in the nucleus of BFTC909 and UM-UC-14 cancer cells. Analysis of CSF-1 intracellular localization by immunofluorescence. Routinely cultured cells were subjected to immunofluorescence using an anti-CSF-1 antibody and nucleus stained with the DAPI. (upper panels: ×400, lower panels: ×200).

To validate the CSF-1 expression, we investigated UTUC tissue samples from 35 patients compared to paired cancer-adjacent normal tissues by immunohistochemistry. We found that the CSF-1 expression was significantly higher in UTUC tissues than in the noncancerous urothelium (*P* = 0.005) (Figure 1(a)). Positive staining expression of CSF-1 predominantly appeared in the nucleus of tumor cells in UTUC tissues (Figure 1(b)). We also used the immunofluorescence method to detect the CSF-1 location in UTUC cell lines (BFTC909 and UM-UC-14). The results revealed that CSF-1 was confined to the cytoplasm and nucleus, and it demonstrated a significantly higher expression in the nucleus than in the cytoplasm (Figure 1(c)).

### 3.2. Association between CSF-1 Expression and Patient Characteristics

**Figure 2:**
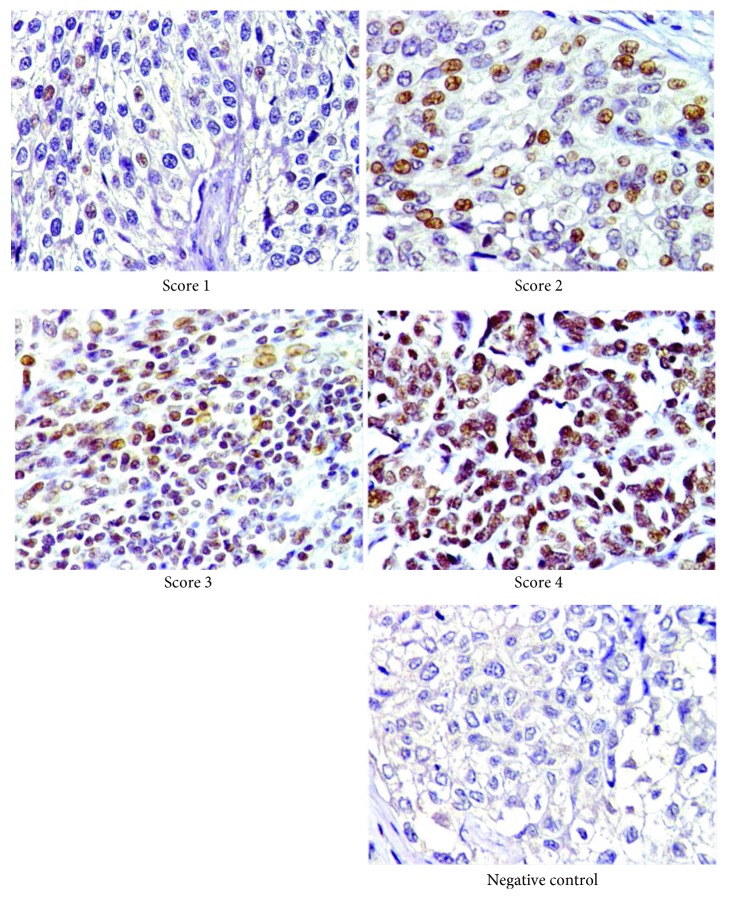
The expression of CSF-1 in UTUC tissue was analyzed by immunohistochemistry. The extent of the expression was partitioned into four classifications: score 1, <25% positive staining of tumor cells; score 2, 26% to 50% positive staining of tumor cells; score 3, 51% to 75% positive staining of tumor cells; and score 4, >76% positive staining of tumor cells (×200).

The expression of CSF-1 in UTUC tissues (*n* = 112) was examined by immunohistochemistry and categorized into four scores (quartiles). On the basis of the scoring, tumor tissues were further sorted into low (scores of 1 and 2; 51.8%) and high (scores of 3 and 4; 48.2%) CSF-1 expression groups (Figure 2 and Table 1). We found that the CSF-1 expression was positively correlated with tumor size (*P* = 0.04, data not shown). Next, we examined the CSF-1 expression for indication of correlation with different clinicopathologic characteristics including tumor stage, grade, gender, age, tumor location, tumor side, lymphovascular invasion, distant metastasis, recurrence, cancer death, and serum creatinine level. The correlations between these clinicopathologic variables and CSF-1 expression are listed in Table 1. We found that high CSF-1 expression in UTUC tissues was significantly associated with tumor stage (*P* < 0.001), distant metastasis (*P* = 0.006), recurrence (*P* = 0.003), and cancer death (*P* = 0.005).

### 3.3. A High Expression of CSF-1 Is Correlated with Poor Prognosis

**Figure 3:**
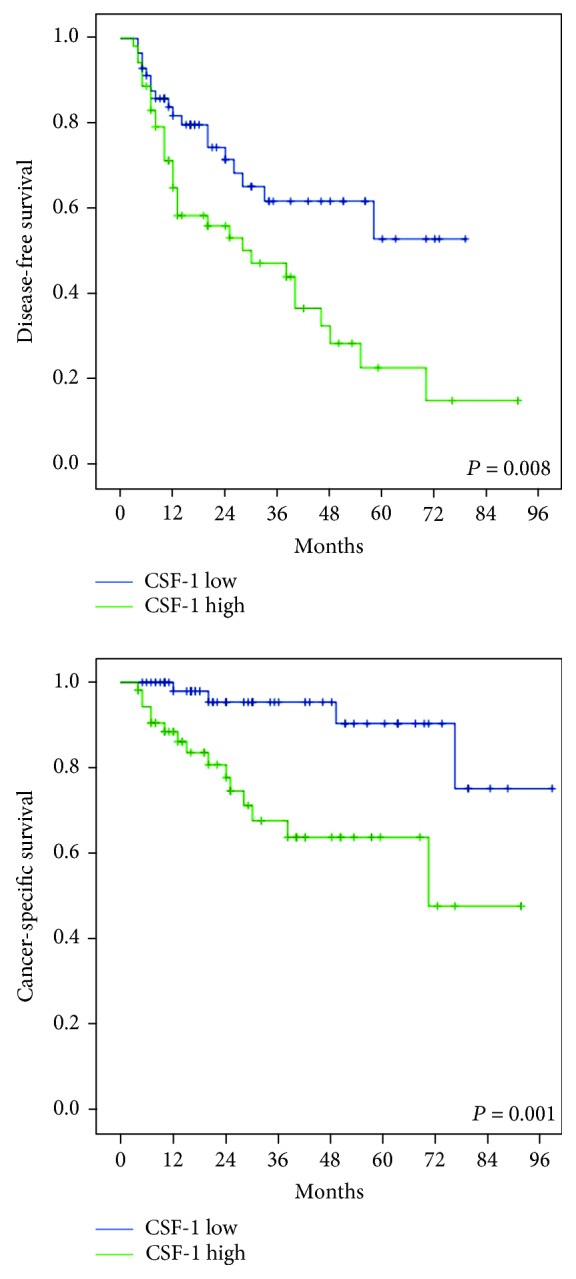
Kaplan-Meier survival curves for disease-free survival and cancer-specific survival rates of patients with CSF-1 expression in upper tract urothelial carcinomas.

To examine parameters related to CSF-1 expression in UTUC patients, we used univariate and multivariate analyses. The data indicated significant associations between disease-free survival and the following two factors: tumor stage (HR = 1.76, CI = 1.01‐3.08, *P* = 0.046) and CSF-1 expression (HR = 2.14, CI = 1.20‐3.81, *P* = 0.01) in univariate analysis (Table 2). However, following the multivariate analysis, only the CSF-1 expression was related to disease-free survival (HR = 2.56, CI = 1.30‐5.04, *P* = 0.007) (Table 2). Univariate analysis also demonstrated that both tumor stage and CSF-1 expression were associated with cancer-specific survival (Table 2). High tumor stage and CSF-1 expression were correlated with a significant reduction in cancer-specific survival (HR = 6.03, CI = 2.17‐16.80, *P* = 0.001, and HR = 5.18, CI = 1.71‐15.71, *P* = 0.004, respectively). In the multivariate analysis, we found that cancer-specific survival was also related to CSF-1 expression (HR = 5.14, CI = 1.27‐20.84, *P* = 0.022). Next, we explored whether the CSF-1 expression in human UTUC tissue samples was correlated to disease-free survival and cancer-specific survival of patients using Kaplan-Meier survival analysis. Kaplan-Meier survival curves showed that higher CSF-1 expression correlated with a significantly lower disease-free survival (*P* = 0.008) and cancer-specific survival (*P* = 0.001) (Figure 3).

## 4. Discussion

We offered the first evidence that high CSF-1 expression is a potential prognostic marker for predicting patient survival and recurrence of UTUC. First, the expression of CSF-1 was higher in UTUC tissues than in cancer-adjacent normal tissues. Second, positive staining of CSF-1 was mainly expressed in the nucleus. Third, a high level of CSF-1 positively correlated with tumor stage, tumor size, distant metastasis, and recurrence. Finally, CSF-1 expression was associated with poor disease-free and cancer-specific survival, and univariate and multivariate proportional hazard analyses indicated that it was also an independent prognostic biomarker for patients with UTUC.

CSF-1 is a cytokine generated by different types of cells, and it regulates the biological functions of monocytes and macrophages, including cell proliferation, differentiation, and survival [16, 17, 31]. Moreover, CSF-1 has also been reported to induce angiogenic activity via recruitment of macrophages, which secrete growth factors, proangiogenic cytokines, and matrix metalloproteases (MMPs) to regulate tumor cell invasion [32]. CSF-1 interacts with CSF-1R, which is a tyrosine kinase transmembrane receptor produced by the *c*-*fms* protooncogene [33]. The CSF-1/CSF-1R axis has an important role in inflammation and immunity [31]. Moreover, CSF-1 and CSF-1R are also expressed in tumor-associated macrophages (TAMs), promoting tumor progression and metastasis in several cancers [18, 34]. Studies have shown that a paracrine loop in CSF-1/CSF-1R signaling between TAMs and tumor cells is required in the tumor microenvironment. Consistent with these findings, our results demonstrated that high expression of CSF-1 in UTUC tissue was correlated with tumor stage and distant metastasis. Furthermore, recent findings indicate that CSF-1 signal transduction pathways have an autocrine-loop function in cancer cells. For instance, the CSF-1/CSF-1R axis could induce phosphorylation and activation of STAT3, which promotes cell survival and proliferation in renal cell carcinoma [35]. Interestingly, our previous studies demonstrated that high activated phospho-STAT3 (Ser727) expression is associated with advanced tumor stage in UTUC tissues and can predict poor prognosis in advanced-stage UTUC patients [15]. STAT3 is a transcription factor whose activation contributes to many cancer functions including survival, proliferation, inflammation, angiogenesis, invasion, and metastasis and is regarded as an oncogene [36–38]. Importantly, STAT3 activation has also been found to contribute to the immunosuppressive tumor microenvironment by prohibiting tumor cell apoptosis and promoting tumor growth and metastasis [39]. Based on the conjunction of previous findings and our studies, we hypothesized that the CSF-1 signaling pathway may be involved in UTUC development by regulating phospho-STAT3 expression. It will be taken into consideration in our future studies.

In this study, the immunohistochemistry analysis revealed that the staining of CSF-1 was primarily expressed in the nucleus, although previous studies indicated the staining position of CSF-1 was also in the cytoplasm of various cancer cells such as renal cell carcinoma, soft tissue sarcomas, and gastric cancer [29, 40, 41]. CSF-1 that is located in the nucleus has been aptly named “nuclear-presenting M-CSF” (nM-CSF) [42, 43]. CSF-1 can also colocalize with CSF-1R in the nucleus in breast cancer cells [44]. Nuclear-presenting M-CSF has been shown to promote cancer cell proliferation and migration [45]. Our immunofluorescence staining in UTUC cancer cells also found CSF-1 to be prominently expressed in the nucleus. The evidence of these studies and our results suggest that CSF-1 expressed in the nucleus may contribute to UTUC progression and metastasis. However, the specific molecular mechanisms of CSF-1 in the nucleus of UTUC cells is not widely understood. Although there was a significant correlation between CSF-1 expression and poor prognosis in this study, the sample size was small; a multi-institutional study with a more substantial sample size is required to verify our results. Finally, we hope our results can help with a prognostic determination for UTUC patients and also help indicate a potential plan for aggressive treatment.

## 5. Conclusions

High CSF-1 expression was found to be an independent predictor of poor survival rates in patients with UTUC. We hope our results will help determine the prognosis for UTUC patients and may also help indicate a plan for aggressive treatment.

## Figures and Tables

**Table 1:** Correlation of CSF-1 expression with clinicopathological characteristics in upper tract urothelial carcinomas.

Variables	Item	Patient no. (%)	CSF-1				*P* value
			Low		High		
			No.	%	No.	%	
Total		112 (100)	58	51.8	54	48.2	

Stage	I/II	73 (65.2)	47	81.0	26	48.1	<0.001^a^
	III/IV	39 (34.8)	11	19.0	28	51.9	

Grade	Low	30 (26.8)	17	29.3	13	24.1	0.532^a^
	High	82 (73.2)	41	70.7	41	75.9	

Gender	Female	68 (60.7)	37	63.8	31	57.4	0.489^a^
	Male	44 (39.3)	21	36.2	23	42.6	

Age (years)	<65	42 (37.5)	17	29.3	25	46.3	0.068^a^
	≥65	70 (62.5)	41	70.7	29	53.7	

Tumor location	Ureter	47 (42.0)	25	43.1	22	40.7	0.300^a^
	Renal pelvis	45 (40.2)	20	34.5	25	46.3	
	Renal pelvis+ureter	20 (17.9)	13	22.4	7	13.0	

Tumor side^c^	Right	49 (44.5)	25	44.6	24	44.4	0.983^a^
	Left	61 (55.5)	31	55.4	30	55.6	

Lymphovascular invasion	Negative	89 (79.5)	48	82.8	41	75.9	0.371^a^
	Positive	23 (20.5)	10	17.2	13	24.1	

Distant metastasis	Negative	96 (85.7)	55	94.8	41	75.9	0.006^b^
	Positive	16 (14.3)	3	5.2	13	24.1	

Recurrence	Negative	62 (55.4)	40	69.0	22	40.7	0.003^a^
	Positive	50 (44.6)	18	31.0	32	59.3	

Cancer death	No	93 (83.0)	54	93.1	39	72.2	0.005^b^
	Yes	19 (17.0)	4	6.9	15	27.8	

Creatinine (mg/dl)	≤1.5	66 (58.9)	35	60.3	31	57.4	0.752^a^
	>1.5	46 (41.1)	23	39.7	23	42.6	

^a^The *P* value was calculated by the chi-square test. ^b^The *P* value was calculated by the Fisher's exact test. ^c^Tumor side was not determined in a small portion of the patients.

**Table 2:** Univariate and multivariate analyses of disease-free survival and cancer-specific survival for upper tract urothelial carcinomas.

Variables	Item	Disease-free survival						Cancer-specific survival					
		Univariate			Multivariate			Univariate			Multivariate		
		HR	95% CI	*P* value	HR	95% CI	*P* value	HR	95% CI	*P* value	HR	95% CI	*P* value
Stage	III/IV	1.76	1.01-3.08	0.046	1.18	0.57-2.44	0.661	6.03	2.17-16.80	0.001	4.18	1.00-17.51	0.051
	I/II	1.00			1.00			1.00			1.00		

Grade	High	1.15	0.61-2.17	0.659	1.05	0.50-2.22	0.902	1.65	0.55-4.99	0.376	0.63	0.14-2.86	0.547
	Low	1.00			1.00			1.00			1.00		

Gender	Male	1.24	0.71-2.16	0.456	1.18	0.64-2.16	0.596	1.36	0.55-3.36	0.501	0.89	0.32-2.43	0.815
	Female	1.00			1.00			1.00			1.00		

Age (years)	≥65	1.68	0.92-3.05	0.091	1.54	0.78-3.07	0.216	1.43	0.54-3.77	0.470	1.11	0.33-3.72	0.870
	<65	1.00			1.00			1.00			1.00		

Tumor location	Renal pelvis + ureter	1.39	0.68-2.83	0.364	1.47	0.64-3.37	0.365	1.11	0.37-3.32	0.856	1.33	0.31-5.82	0.701
	Renal pelvis	0.76	0.40-1.45	0.406	0.67	0.34-1.31	0.243	0.56	0.19-1.68	0.301	0.42	0.13-1.34	0.143
	Ureter	1.00			1.00			1.00			1.00		

Tumor side	Left	0.80	0.46-1.40	0.443	0.84	0.46-1.55	0.584	0.59	0.24-1.48	0.263	0.43	0.14-1.29	0.131
	Right	1.00			1.00			1.00			1.00		

Lymphovascular invasion	Positive	1.35	0.72-2.55	0.351	1.13	0.54-2.39	0.745	2.49	0.97-6.34	0.057	2.15	0.69-6.70	0.186
	Negative	1.00			1.00			1.00			1.00		

Creatinine (mg/dl)	>1.5	1.06	0.60-1.88	0.844	0.93	0.50-1.73	0.822	0.76	0.29-2.00	0.570	0.59	0.19-1.76	0.342
	≤1.5	1.00			1.00			1.00			1.00		

CSF-1	High	2.14	1.20-3.81	0.010	2.56	1.30-5.04	0.007	5.18	1.71-15.71	0.004	5.14	1.27-20.84	0.022
	Low	1.00			1.00			1.00			1.00		

Abbreviations: HR: hazard ratio; CI: confidence interval.

## Data Availability

The data used to support the findings of this study are included within the article.
